# An Algorithm for the Mixed Transportation Network Design Problem

**DOI:** 10.1371/journal.pone.0162618

**Published:** 2016-09-14

**Authors:** Xinyu Liu, Qun Chen

**Affiliations:** 1 School of Traffic and Transportation Engineering, Central South University, Changsha, 410075, China; 2 School of Business & Law, Foshan University, Foshan, 528000, China; Southwest University, CHINA

## Abstract

This paper proposes an optimization algorithm, the dimension-down iterative algorithm (DDIA), for solving a mixed transportation network design problem (MNDP), which is generally expressed as a mathematical programming with equilibrium constraint (MPEC). The upper level of the MNDP aims to optimize the network performance via both the expansion of the existing links and the addition of new candidate links, whereas the lower level is a traditional Wardrop user equilibrium (UE) problem. The idea of the proposed solution algorithm (DDIA) is to reduce the dimensions of the problem. A group of variables (discrete/continuous) is fixed to optimize another group of variables (continuous/discrete) alternately; then, the problem is transformed into solving a series of CNDPs (continuous network design problems) and DNDPs (discrete network design problems) repeatedly until the problem converges to the optimal solution. The advantage of the proposed algorithm is that its solution process is very simple and easy to apply. Numerical examples show that for the MNDP without budget constraint, the optimal solution can be found within a few iterations with DDIA. For the MNDP with budget constraint, however, the result depends on the selection of initial values, which leads to different optimal solutions (i.e., different local optimal solutions). Some thoughts are given on how to derive meaningful initial values, such as by considering the budgets of new and reconstruction projects separately.

## Introduction

### Definition of network design problem (NDP)

The NDP is concerned with modifying a transportation network configuration by adding new links or improving the existing ones to maximize certain social welfare objectives (e.g., total travel time over the network). How to select the location of these new links and how much additional capacity is to be added to each of these existing links are motivating problems and involve trying to minimize the total system costs under a limited budget while accounting for the route choice behavior of network users.

The NDP can be generally formulated as a mathematical programming with equilibrium constraint (MPEC) problem. A deterministic user equilibrium assignment model (UE) or stochastic user equilibrium assignment model (SUE) is usually applied to describe the route choice behavior of network users. Various solution algorithms, including gradient-based and derivative-free (or meta-) heuristic algorithms, have been proposed for solving the family of NDPs [1–3].

### Discrete network design problem (DNDP), continuous network design problem (CNDP), and mixed network design problem (MNDP)

The NDPs can be roughly classified into three categories: the discrete network design problem (DNDP), which addresses the selection of the optimal locations (expressed by 0–1 integer decision variables) of new links to be added; the continuous network design problem (CNDP), which determines the optimal capacity enhancement (expressed by continuous decision variable) for a subset of existing links; and the mixed network design problem (MNDP), which combines CNDP and DNDP in a single network.

The CNDP has been widely studied because of the continuousness of variables (capacity decisions are supposed to be continuous), which allows the use of many different modeling approaches and solution methodologies [4–7]. The body of DNDP literature is somewhat smaller than that of CNDP, probably because of the complexity resulting from the presence of discrete variables. Exact methods such as branch and bound can be found mostly in DNDP. Examples of the use of such methods can be found in the literature [8–11]. They developed the branch and bound algorithm to directly solve their (upper) problems.

The MNDP involves both discrete and continuous variables and can be generally expressed as a nonlinear mixed-integer bi-level programming, which is normally difficult to solve [1]. Very few studies in this field have been accomplished over the last decade. Some solution algorithms for MNDPs can be categorized as heuristic or meta-heuristic [12–14]. The literature [15] has proposed a global optimization algorithm for solving the MNDP, in which the UE condition is formulated as a variational inequality (VI) problem. The MNDP is approximated as a piecewise-linear programming (P-LP) problem and is then transformed into a mixed-integer linear programming (MILP) problem. However, the linearization process of the link impedance function may be time-consuming and may introduce errors.

Table 1 gives an updated summary of the existing algorithms for the three network design problems mentioned above.

**Table 1:** Algorithms for solving NDPs.

Problem	Name of the algorithm	Sources
CNDP	*Gradient-based algorithms*	
	Sensitivity analysis-based method	[16–25]
	Gradient Projection method	[6]
	Augmented Lagrangian algorithm	[5]
	*Derivative-free (meta-) heuristic algorithms*	
	Iterative optimization-assignment algorithm	[26–29]
	Hooke–Jeeves algorithm	[30]
	Equilibrium decomposed optimization	[31]
	Genetic algorithm	[32]
	Simulated annealing algorithm	[4]
	Path based mixed-integer linear program	[33]
	*Global optimization method*	[3, 7]
DNDP	Branch-and-bound techniques	[8, 9–11, 34]
	Lagrange relaxation and dual ascent process	[35]
	Decomposition with quasi-optimization	[36]
	Support function concept	[37]
	Ant system method	[38]
	Genetic algorithm	[39–40]
	Hybrid meta-heuristic algorithm	[41]
	Parsimonious heuristic	[42]
	Global optimization methods	[43–45]
MNDP	mixed-integer linear programming approach	[15]
	Hill Climbing, Simulated Annealing, Tabu Search, Genetic Algorithm, Hybrids of Tabu Search	[12,13]
	Scatter Search	[14]

### Motivation of this study

This paper attempts to develop an algorithm for solving the MNDP, in which the idea is to reduce the dimensions of the problem. The solution of the MNDP is changed to the iterative solution of some CNDPs and DNDPs until it converges to an optimal solution. The principle of the proposed algorithm is very simple and can be easily applied. Moreover, the existing algorithms for CNDPs and DNDPs can be directly used in the iterative solution for MNDPs, thus eliminating the need to design special algorithms for MNDPs. Numerical examples are presented to demonstrate the efficiency of the proposed iterative method through comparisons with other algorithms reported in the literature.

The remainder of the paper is organized as follows. Section 2 presents some basic components of the MNDP. Section 3 proposes the dimension-down iterative algorithm (DDIA) for solving the MNDP. In Section 4, numerical experiments and comparisons of the results with those of the previous algorithms are given. The final section concludes the paper.

## Problem Formulation

### Notations

The notations used throughout the paper are listed as follows unless otherwise specified.

**G** = (**N, A**) a transportation network with **N** being the set of nodes and **A** (**A** = **A**_**1**_∪**A**_**2**_∪**A**_**3**_**)** being the set of links

**R** set of origin nodes, where **R⊂N**

**S** set of destination nodes, where **S⊂N**

*r* origin node index, where *r***∈R**

*s* destination node index, where *s***∈S**

A_1_ set of non-expanded links, where A_1_ ⊂ A

A_2_ set of expanded links, where A_2_ ⊂ A

A_3_ set of new candidate links, where A_3_ ⊂ A

*x*_*a*_ aggregate flow on link *a* ∈ A

**x** vector whose elements are *x*_*a*_

$y_{a}^{0}$ original capacity on existing link *a* ∈ A_1_ ∪ A_2_

*y*_*a*_ incremental capacity on expanded link *a* ∈ A_2_

**y** vector whose elements are *y*_*a*_

$y_{a}^{\prime }$ fixed capacities on new candidate link *a* ∈ A_3_

*t*_*a*_ travel time of link *a* ∈ A

*g*_*a*_(*y*_*a*_) improvement cost function of expanded link *a* ∈ A_2_

*c*_*a*_ improvement cost per unit incremental capacity of expanded link *a* ∈ A_2_

*d*_*a*_ construction cost per addition of new candidate link *a* ∈ A_3_

*u*_*a*_ 0–1 decision variable, *u*_*a*_ = 1 if link *a* ∈ A_3_ is added, otherwise *u*_*a*_ = 0

u vector whose elements are *u*_*a*_

*ϕ* relative weight of construction cost and travel time

*qrs* travel demand between pair (*r*, *s*)

*fkrs* flow of path *k* between pair (*r*,*s*)

**L**_*rs*_ set of paths between pair (*r*,*s*)

$\delta_{a,k}^{rs}$ path/link incidence variable, which equals 1 if link *a* is on path *k* between pair (*r*,*s*) and is 0 otherwise

DDIA dimension-down iterative algorithm

### Formulations of DNDP, CNDP and MNDP

#### Formulation of DNDP

The discrete network design problem (DNDP) addresses only the selection of the optimal locations (expressed by 0–1 integer decision variables) of new links to be added. The DNDPs include two categories: the DNDP with budget constraint and the DNDP without budget constraint [8, 9, 34, 37].

**(the DNDP without budget constraint)**

$$\underset{\text{u}}{\text{min}}Z(\text{u,x})=\sum_{a\in A_{1}\cup {\text{A}}_{2}}x_{a}t_{a}(x_{a},y_{a}^{0})+\sum_{a\in {\text{A}}_{3}}x_{a}t_{a}(x_{a},y_{a}^{\prime })+\phi \sum_{a\in {\text{A}}_{3}}d_{a}u_{a}$$ (1)

s.t.

$$u_{a}=\{0,1\},\;\forall a\in {\text{A}}_{3}$$ (2)

where **x** is an implicit function of **u** and can be obtained by solving the lower-level UE problem [46].

$$\text{min}T(\text{u,x})=\sum_{a\in {\text{A}}_{1}\cup {\text{A}}_{2}}\int_{\text{0}}^{x_{a}}t_{a}(w,y_{a}^{0})dw+\sum_{a\in {\text{A}}_{3}}\int_{\text{0}}^{x_{a}}t_{a}(w,y_{a}^{\prime })dw$$ (3)

s.t.

$$\sum_{k\in {\text{L}}_{rs}}f_{k}^{rs}=q_{rs},\;\forall r\in \text{R},\;s\in \text{S}$$ (4)

$$x_{a}=\sum_{r,s}\sum_{k}f_{k}^{rs}\cdot \delta_{a,k}^{rs}\;\forall a\in \text{A}$$ (5)

$$f_{k}^{rs}\geq 0,\;\forall r\in \text{R},\;s\in \text{S, }k\in {\text{L}}_{rs}$$ (6)

**(the DNDP with budget constraint)**

$$\underset{\text{u}}{\text{min}}Z(\text{u,x})=\sum_{a\in A_{1}\cup {\text{A}}_{2}}x_{a}t_{a}(x_{a},y_{a}^{0})+\sum_{a\in {\text{A}}_{3}}x_{a}t_{a}(x_{a},y_{a}^{\prime })$$ (7)

s.t.

$$\sum_{a\in {\text{A}}_{3}}d_{a}u_{a}\leq budget$$ (8)

$$u_{a}=\{0,1\},\;\forall a\in {\text{A}}_{3}$$ (9)

where **x** is an implicit function of **u** and can be obtained by solving the lower-level problem (formulas (3)~(6)).

#### Formulation of CNDP

The continuous network design problem (CNDP) determines the optimal capacity enhancement for a subset of existing links; its decision variables are continuous. A_3_ = Φ because there are no new candidate links to be added. The CNDPs have two types: the CNDP with budget constraint and the CNDP without budget constraint [23, 24, 30, 31, 33].

**(the CNDP without budget constraint)**

$$\underset{\text{y}}{\text{min}}Z(\text{y,x})=\sum_{a\in {\text{A}}_{1}}x_{a}t_{a}(x_{a})+\sum_{a\in {\text{A}}_{2}}x_{a}t_{a}(x_{a},y_{a})+\phi \sum_{a\in {\text{A}}_{2}}g_{a}(y_{a})$$ (10)

s.t.

$$y_{a}^{0}\leq y_{a}\leq {\bar{y}}_{a},\;\forall a\in {\text{A}}_{2}$$ (11)

where ${\bar{y}}_{a}$ is the upper bound of the incremental capacity on the expanded link *a* ∈ A_2_. **x** is an implicit function of **y** and can be obtained by solving the lower-level problem.

$$\text{min}T(\text{y,x})=\sum_{a\in {\text{A}}_{1}}\int_{\text{0}}^{x_{a}}t_{a}(w)dw+\sum_{a\in {\text{A}}_{2}}\int_{\text{0}}^{x_{a}}t_{a}(w,y_{a})dw$$ (12)

s.t.

$$\sum_{k\in {\text{L}}_{rs}}f_{k}^{rs}=q_{rs},\;\forall r\in \text{R},\;s\in \text{S}$$ (13)

$$x_{a}=\sum_{r,s}\sum_{k}f_{k}^{rs}\cdot \delta_{a,k}^{rs}\;\forall a\in \text{A}$$ (14)

$$f_{k}^{rs}\geq 0,\;\forall r\in \text{R},\;s\in \text{S, }k\in {\text{L}}_{rs}$$ (15)

**(the CNDP with budget constraint)**

$$\underset{\text{y}}{\text{min}}Z(\text{y,x})=\sum_{a\in {\text{A}}_{1}}x_{a}t_{a}(x_{a})+\sum_{a\in {\text{A}}_{2}}x_{a}t_{a}(x_{a},y_{a})$$ (16)

s.t.

$$\sum_{a\in {\text{A}}_{2}}g_{a}(y_{a})\leq budget$$ (17)

$$y_{a}^{0}\leq y_{a}\leq {\bar{y}}_{a},\;\forall a\in {\text{A}}_{2}$$ (18)

where **x** is an implicit function of **y** and can be obtained by solving the lower-level problem (formulas (12)~(15)).

#### Formulation of MNDP

The MNDP aims to find both capacity expansions of existing links (continuous decision variables) and new link additions (0–1 decision variables) to minimize the total travel time of the network users subject to a budgetary constraint and the UE condition [1, 14, 15]. The MNDP is formulated as

**(the MNDP without budget constraint)**

$$\underset{\text{y,u}}{\text{min}}Z(\text{y,u,x})=\sum_{a\in {\text{A}}_{1}}x_{a}t_{a}(x_{a})+\sum_{a\in {\text{A}}_{2}}x_{a}t_{a}(x_{a},y_{a})+\sum_{a\in {\text{A}}_{3}}x_{a}t_{a}(x_{a},y_{a}^{\prime })+\phi \sum_{a\in {\text{A}}_{2}}g_{a}(y_{a})+\phi \sum_{a\in {\text{A}}_{3}}d_{a}u_{a}$$ (19)

s.t.

$$y_{a}^{0}\leq y_{a}\leq {\bar{y}}_{a},\;\forall a\in {\text{A}}_{2}$$ (20)

$$u_{a}=\{0,1\},\;\forall a\in {\text{A}}_{3}$$ (21)

where **x** is an implicit function of **y** and **u** and can be obtained by solving the lower-level problem.

$$\text{min}T(\text{y,u,x})=\sum_{a\in {\text{A}}_{1}}\int_{\text{0}}^{x_{a}}t_{a}(w)dw+\sum_{a\in {\text{A}}_{2}}\int_{\text{0}}^{x_{a}}t_{a}(w,y_{a})dw+\sum_{a\in {\text{A}}_{3}}\int_{\text{0}}^{x_{a}}t_{a}(w,y_{a}^{\prime })dw$$ (22)

s.t.

$$\sum_{k\in {\text{L}}_{rs}}f_{k}^{rs}=q_{rs},\;\forall r\in \text{R},\;s\in \text{S}$$ (23)

$$x_{a}=\sum_{r,s}\sum_{k}f_{k}^{rs}\cdot \delta_{a,k}^{rs}\;\forall a\in \text{A}$$ (24)

$$f_{k}^{rs}\geq 0,\;\forall r\in \text{R},\;s\in \text{S},\;k\in {\text{L}}_{rs}$$ (25)

**(the MNDP with budget constraint)**

$$\underset{\text{y,u}}{\text{min}}Z(\text{y,u,x})=\sum_{a\in {\text{A}}_{1}}x_{a}t_{a}(x_{a})+\sum_{a\in {\text{A}}_{2}}x_{a}t_{a}(x_{a},y_{a})+\sum_{a\in {\text{A}}_{3}}x_{a}t_{a}(x_{a},y_{a}^{\prime })$$ (26)

s.t.

$$\sum_{a\in {\text{A}}_{2}}g_{a}(y_{a})+\sum_{a\in {\text{A}}_{3}}d_{a}u_{a}\leq budget$$ (27)

$$y_{a}^{0}\leq y_{a}\leq {\bar{y}}_{a},\;\forall a\in {\text{A}}_{2}$$ (28)

$$u_{a}=\{0,1\},\;\forall a\in {\text{A}}_{3}$$ (29)

where **x** is an implicit function of **y** and **u** and can be obtained by solving the lower-level problem (formulas (22)~(25)).

## Dimension-Down Iterative Algorithm (DDIA) for Solving the MNDP

The idea of the dimension-down iterative algorithm (DDIA) is to reduce the dimensions of the problem. A group of variables (discrete/continuous) is fixed to optimize another group of variables (continuous/discrete) alternately; then, the problem is transformed to solve a series of CNDPs and DNDPs repeatedly until it converges to the optimal solution.

Suppose u^(0)^ = {$u_{1}^{\left(0\right)}$, $u_{2}^{\left(0\right)}$, $u_{3}^{\left(0\right)}$, ……} is a feasible solution within the budget constraint (e.g., let u^(0)^ = (0, 0, 0, ……, 0)), **u** is fixed at **u**^(0)^ to optimize **y**; therefore, the problem becomes solving a CNDP. The methods for solving the CNDP listed in Table 1 can be applied to solve this problem.

$$\underset{{\text{y,u}}^{\text{(0)}}}{\text{min}}Z({\text{y,u}}^{\text{(0)}}\text{,x})=\sum_{a\in {\text{A}}_{1}}x_{a}t_{a}(x_{a})+\sum_{a\in {\text{A}}_{2}}x_{a}t_{a}(x_{a},y_{a})+\sum_{a\in {\text{A}}_{3}}x_{a}t_{a}(x_{a},y_{a}^{\prime })+\phi \sum_{a\in {\text{A}}_{2}}g_{a}(y_{a})+\phi \sum_{a\in {\text{A}}_{3}}d_{a}u_{a}^{\left(0\right)}$$ (30)

s.t.

$$y_{a}^{0}\leq y_{a}\leq {\bar{y}}_{a},\;\forall a\in {\text{A}}_{2}$$ (31)

where **x** is an implicit function of **y** and can be obtained by solving the lower-level problem.

$$\text{min}T({\text{y,u}}^{\text{(0)}}\text{,x})=\sum_{a\in {\text{A}}_{1}}\int_{\text{0}}^{x_{a}}t_{a}(w)dw+\sum_{a\in {\text{A}}_{2}}\int_{\text{0}}^{x_{a}}t_{a}(w,y_{a})dw+\sum_{a\in {\text{A}}_{3}}\int_{\text{0}}^{x_{a}}t_{a}(w,y_{a}^{\prime })dw$$ (32)

s.t.

$$\sum_{k\in {\text{L}}_{rs}}f_{k}^{rs}=q_{rs},\;\forall r\in \text{R},\;s\in \text{S}$$ (33)

$$x_{a}=\sum_{r,s}\sum_{k}f_{k}^{rs}\cdot \delta_{a,k}^{rs}\;\forall a\in \text{A}$$ (34)

$$f_{k}^{rs}\geq 0,\;\forall r\in \text{R},\;s\in \text{S, }k\in {\text{L}}_{rs}$$ (35)

If there is a budget constraint, the problem to be solved is as follows.

$$\underset{{\text{y,u}}^{\text{(0)}}}{\text{min}}Z({\text{y,u}}^{\text{(0)}}\text{,x})=\sum_{a\in {\text{A}}_{1}}x_{a}t_{a}(x_{a})+\sum_{a\in {\text{A}}_{2}}x_{a}t_{a}(x_{a},y_{a})+\sum_{a\in {\text{A}}_{3}}x_{a}t_{a}(x_{a},y_{a}^{\prime })$$ (36)

s.t.

$$\sum_{a\in {\text{A}}_{2}}g_{a}(y_{a})+\sum_{a\in {\text{A}}_{3}}d_{a}u_{a}^{\left(0\right)}\leq budget$$ (37)

$$y_{a}^{0}\leq y_{a}\leq {\bar{y}}_{a},\;\forall a\in {\text{A}}_{2}$$ (38)

where **x** is an implicit function of **y** and can be obtained by solving the lower-level problem (formulas (32)~(35)).

Solve the above CNDP and obtain the solution **y**^(0)^ = {$y_{1}^{\left(0\right)}$, $y_{2}^{\left(0\right)}$, $y_{3}^{\left(0\right)}$, ……}. Then, **y** is fixed at **y**^(0)^ to optimize **u.**

$$\underset{{\text{y}}^{\text{(0)}}\text{,u}}{\text{min}}Z({\text{y}}^{\text{(0)}}\text{,u,x})=\sum_{a\in {\text{A}}_{1}}x_{a}t_{a}(x_{a})+\sum_{a\in {\text{A}}_{2}}x_{a}t_{a}(x_{a},y_{a}^{\left(0\right)})+\sum_{a\in {\text{A}}_{3}}x_{a}t_{a}(x_{a},y_{a}^{\prime })+\phi \sum_{a\in {\text{A}}_{2}}g_{a}(y_{a}^{\left(0\right)})+\phi \sum_{a\in {\text{A}}_{3}}d_{a}u_{a}$$ (39)

s.t.

$$u_{a}=\{0,1\},\;\forall a\in {\text{A}}_{3}$$ (40)

where **x** is an implicit function of **u** and can be obtained by solving the lower-level problem.

$$\text{min}T({\text{y}}^{\text{(0)}}\text{,u,x})=\sum_{a\in {\text{A}}_{1}}\int_{\text{0}}^{x_{a}}t_{a}(w)dw+\sum_{a\in {\text{A}}_{2}}\int_{\text{0}}^{x_{a}}t_{a}(w,y_{a}^{\left(0\right)})dw+\sum_{a\in {\text{A}}_{3}}\int_{\text{0}}^{x_{a}}t_{a}(w,y_{a}^{\prime })dw$$ (41)

s.t.

$$\sum_{k\in {\text{L}}_{rs}}f_{k}^{rs}=q_{rs},\;\forall r\in \text{R},\;s\in \text{S}$$ (42)

$$x_{a}=\sum_{r,s}\sum_{k}f_{k}^{rs}\cdot \delta_{a,k}^{rs}\;\forall a\in \text{A}$$ (43)

$$f_{k}^{rs}\geq 0,\;\forall r\in \text{R},\;s\in \text{S},\;k\in {\text{L}}_{rs}$$ (44)

If there is a budget constraint, the problem to be solved is as follows.

$$\underset{{\text{y}}^{\text{(0)}}\text{,u}}{\text{min}}Z({\text{y}}^{\text{(0)}}\text{,u,x})=\sum_{a\in {\text{A}}_{1}}x_{a}t_{a}(x_{a})+\sum_{a\in {\text{A}}_{2}}x_{a}t_{a}(x_{a},y_{a}^{\left(0\right)})+\sum_{a\in {\text{A}}_{3}}x_{a}t_{a}(x_{a},y_{a}^{\prime })$$ (45)

s.t.

$$\sum_{a\in {\text{A}}_{2}}g_{a}(y_{a}^{\left(0\right)})+\sum_{a\in {\text{A}}_{3}}d_{a}u_{a}\leq budget$$ (46)

$$u_{a}=\{0,1\},\;\forall a\in {\text{A}}_{3}$$ (47)

where **x** is an implicit function of **u** and can be obtained by solving the lower-level problem (formulas (41)~(44)).

The above problem is to solve a DNDP, and the methods for solving the DNDP listed in Table 1 can be applied. Suppose the solution is **u**^(1)^ = {$u_{1}^{\left(1\right)}$, $u_{2}^{\left(1\right)}$, $u_{3}^{\left(1\right)}$, ……}, then **u** is fixed at **u**^(1)^ to optimize **y.** In this way, a series of solutions {**u**^(k)^}, {**y**^(k)^} (k = 0, 1, 2, ……) can be obtained.

Because

$$Z({\text{y,u}}^{\text{(0)}}\text{,x})\;\geq Z({\text{y}}^{\text{(0)}}{\text{,u}}^{\text{(0)}}\text{,x})\;\geq \;Z({\text{y}}^{\text{(0)}}{\text{,u}}^{\text{(1)}}\text{,x})\;\geq \cdots Z({\text{y}}^{\text{(k)}}{\text{,u}}^{\text{(k)}}\text{,x})\;\geq \cdots \geq \;Z_{\text{min}}$$ (48)

where *Z*_min_ is the optimal objective function value. Only if an optimal solution exists for the MNDPs (formulas (19)~(25) or (26)~(29)), does *Z*_min_ (the lower bound of *Z*) exist. Therefore, the function value series {*Z*(y^(k)^, u^(k)^, x)} is monotonically decreasing, but it attains *Z*_min_ at most. That is, it always converges but may converge to a value larger than *Z*_min_ (namely a local optimal solution, and the quality of the solution depends on the applied algorithms for CNDPs and DNDPs). Therefore, several initial values should be tested, and the best solution can be selected from among them.

## Numerical Examples

### A simple test network

**Fig 1:**
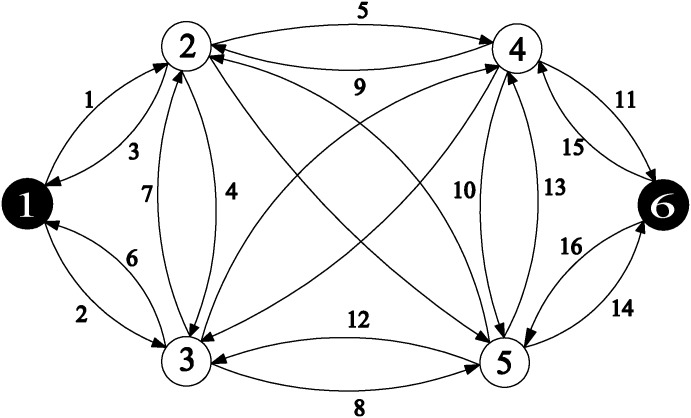
A simple test network.

A simple test network is given (Fig 1). Links 1~16 are expanded links, and links 17, 18, 19, and 20 are new candidate links. The link parameters are listed in Table 2 and OD matrix is indicated in Table 3. Let *ϕ* = 1 and the investment function *ga*(*ya*) = *caya*.

**Table 2:** Link parameters for simple test network.

$t_{a}=t_{a}(x_{a},y_{a})=\alpha_{a}+\beta_{a}{\left(x_{a}/(y_{a}^{0}+y_{a})\right)}^{4},\;a\in {\text{A}}_{2}$;$t_{a}=t_{a}(x_{a},y_{a}^{\prime })=\alpha_{a}+\beta_{a}{\left(x_{a}/y_{a}^{\prime }\right)}^{4},\;a\in {\text{A}}_{3}$; $Z=\sum_{a\in \text{A}}x_{a}t_{a}+\sum_{a\in {\text{A}}_{2}}c_{a}y_{a}+\sum_{a\in {\text{A}}_{3}}d_{a}u_{a}$							
link	node		$y_{a}^{0}$ or $y_{a}^{\prime }$	*α*_*a*_	*β*_*a*_	*c*_*a*_ or *d*_*a*_	y or u
	*i*	*j*					
1	1	2	3	1	10	2	*y*_1_
2	1	3	10	2	5	3	*y*_2_
3	2	1	9	3	3	5	*y*_3_
4	2	3	4	4	20	4	*y*_4_
5	2	4	3	5	50	9	*y*_5_
6	3	1	2	2	20	1	*y*_6_
7	3	2	1	1	10	4	*y*_7_
8	3	5	10	1	1	3	*y*_8_
9	4	2	45	2	8	2	*y*_9_
10	4	5	3	3	3	5	*y*_10_
11	4	6	2	9	2	6	*y*_11_
12	5	3	6	4	10	8	*y*_12_
13	5	4	44	4	25	5	*y*_13_
14	5	6	20	2	33	3	*y*_14_
15	6	4	1	5	5	6	*y*_15_
16	6	5	4.5	6	1	1	*y*_16_
17	3	4	26	4	9	8	*u*_1_
18	4	3	21	3	15	9	*u*_2_
19	2	5	35	3	11	10	*u*_3_
20	5	2	41	4	8	6	*u*_4_

**Table 3:** OD matrix for simple test network.

Node	**1**	**2**	**3**	**4**	**5**	**6**
**1**	0	0	0	3	2	5
**2**	0	0	0	0	0	4
**3**	0	0	0	0	0	0
**4**	0	0	0	0	0	0
**5**	2	0	0	0	0	0
**6**	10	4	3	0	0	0

The objective function value of each iteration and the optimal solution of MNDP obtained from the proposed solution algorithm (DDIA) are presented in Table 4. Note that here the DNDP is solved by the enumeration method while the Hooke-Jeeves algorithm is applied for solving the CNDP.

**Table 4:** Solution of simple test network.

Iteration		Fixed value	Solution	*Z*
1	solving CNDP	u^(0)^ = (0 0 0 0)	y^(0)^ = (0 2.5625 4.0000 3.0000 0 0 0 3.3750 0 0 0 0 0 0 0.1250 16.3750)	474.9184
2	solving DNDP	y^(0)^ = (0 2.5625 4.0000 3.0000 0 0 0 3.3750 0 0 0 0 0 0 0.1250 16.3750)	u^(1)^ = (0 0 1 1)	424.9987
3	solving CNDP	u^(1)^ = (0 0 1 1)	y^(1)^ = (1.5625 1.1250 3.6875 0 0 0.7500 0 0 0 0 0 0 0 0 0 15.1875)	403.3460
4	solving DNDP	y^(1)^ = (1.5625 1.1250 3.6875 0 0 0.7500 0 0 0 0 0 0 0 0 0 15.1875)	u^(2)^ = (0 0 1 1)	403.346; *convergence*

To determine whether the proposed solution algorithm (DDIA) has found the optimal solution of the MNDP for this test network, the new candidate links 17, 18, 19, and 20 are combined to obtain 16 total possible combinations. We solve a corresponding CNDP for each combination (Table 5).

**Table 5:** Solution under the fixed u.

u	y	*Z*
(0 0 0 0)	(0 2.5625 4.0000 3.0000 0 0 0 3.3750 0 0 0 0 0 0 0.1250 16.3750)	474.9184
(1 0 0 0)	(0 3.1250 4.2500 3.3125 0 0 0 1.6250 0 0 0 0 0 0 0.1250 16.6250)	471.2424
(0 1 0 0)	(0 3.1875 1.7500 3.3125 0 6.5000 0 3.8125 0 0 0 0 0 0 0 16.2500)	473.0195
(0 0 1 0)	(1.6250 1.0000 4.2500 0 0 0 0 0 0 0 0 0 0 0 0 16.5625)	437.4500
(0 0 0 1)	(0 2.8750 4.3125 2.8750 0 0 0 3.3750 0 0 0 0 0 0 0 16.8750)	440.6147
(1 1 0 0)	(0 3.0625 0.7500 3.3125 0 8.5625 0 1.6250 0 0 0 0 0 0 0 16.5625)	465.3606
(1 0 1 0)	(0 2.8125 4.1250 0 0 0 0 0 0 0 0 0 0 0 0 16.6250)	439.0100
(1 0 0 1)	(0 3.2500 4.3750 3.3125 0 0.1250 0 1.6250 0 0 0 0 0 0 0 16.7500)	439.9451
(0 1 1 0)	(3.0000 0.2500 0.1875 0 0 7.6250 0 0 0 0 0 0 0 0 0.1875 16.5625)	431.0058
(0 1 0 1)	(0 2.0625 0.7500 3.3125 0 7.7500 0 3.7500 0 0 0 0 0 0 3.50 12.750)	450.2416
**(0 0 1 1)**	**(1.5625 1.1250 3.6875 0 0 0.7500 0 0 0 0 0 0 0 0 0 15.1875)**	**403.3460**
(0 1 1 1)	(3.1250 0.1250 0.8750 0 0 9.1250 0 0 0 0 0 0 0 0 3.2500 11.1250)	414.8057
(1 0 1 1)	(0.6875 1.5000 3.3750 0 0 1.5000 0 0 0 0 0 0 0 0 0 16.5000)	406.1308
(1 1 0 1)	(0.1875 2.8750 2.0000 3.3125 0 6.7500 0 1.6250 0 0 0 0 0 0 0 16.6250)	444.4567
(1 1 1 0)	(2.1250 0.6250 2.3750 0 0 4.2500 0 0 0 0 0 0 0 0 0 16.5625)	437.6232
(1 1 1 1)	(1.0000 1.5000 1.7500 0 0 5.7500 0 0 0 0 0 0 0 0 0 17.7500)	410.8002

In Table 5, the solution with a minimal objective function value is **u** = (0 0 1 1), **y** = (1.5625 1.1250 3.6875 0 0 0.7500 0 0 0 0 0 0 0 0 0 15.1875), *Z =* 403.3460, which is consistent with the solution found by the proposed solution algorithm (DDIA).

### The Sioux Falls network

**Fig 2:**
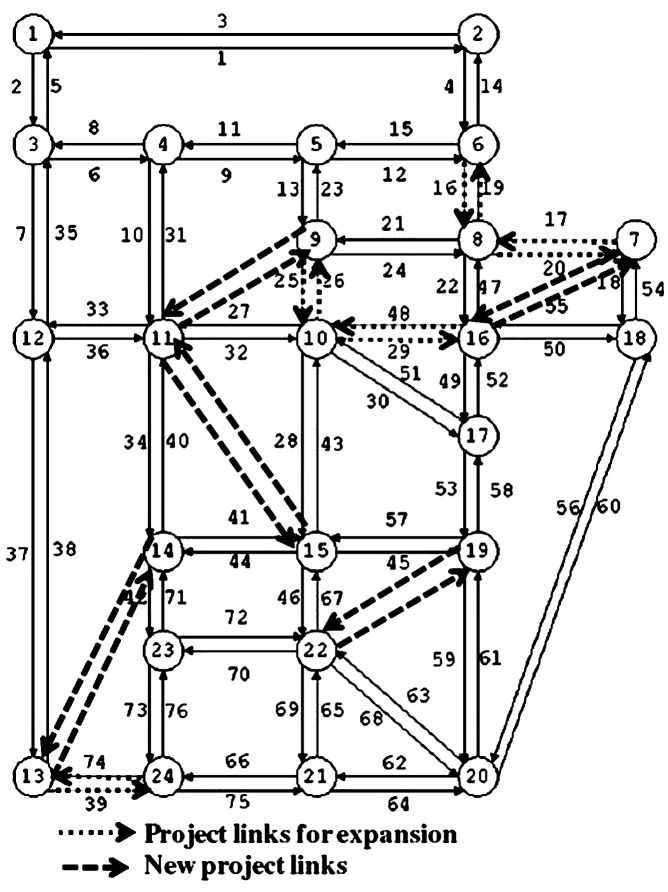
Sioux Falls network.

The network and all input information are the same as those used in [15]. The link data and the O-D travel demands between 552 O-D pairs are presented in S1 and S2 Tables, respectively. For the MNDP, there are ten candidate projects; five are for capacity improvements of the existing links (represented by dotted lines in Fig 2), and the other five involve constructions of new links (represented by dashed lines).

The objective function value of each iteration and the optimal solution of MNDP obtained from the proposed solution algorithm (DDIA) are presented in Table 6. Note that here the DNDP is solved using the enumeration method, and the Hooke-Jeeves algorithm is applied to solve the CNDP.

**Table 6:** Solution of the Sioux Falls network.

Iteration		Fixed value	Solution	*Z*
1	solving CNDP	u^(0)^ = (0 0 0 0 0)	y^(0)^ = (5.2500 2.7500 5.2500 2.2500 3.2500 1.9375 3.0000 5.0000 3.5000 5.0000)	81.4238
2	solving DNDP	y^(0)^ = (5.2500 2.7500 5.2500 2.2500 3.2500 1.9375 3.0000 5.0000 3.5000 5.0000)	u^(1)^ = (0 1 1 1 1)	68.7179
3	solving CNDP	u^(1)^ = (0 1 1 1 1)	y^(1)^ = (3.2500 1.2500 3.7500 1.2500 0.7500 0.9375 4.2500 1.0000 4.5000 1.0000)	66.8700
4	solving DNDP	y^(1)^ = (3.2500 1.2500 3.7500 1.2500 0.7500 0.9375 4.2500 1.0000 4.5000 1.0000)	u^(2)^ = (0 1 1 1 1)	66.870; *convergence*

From the above calculation, we can see that the convergence speed is high; it can converge within a few iterations. This may occur because the number of variables is very small. If there are more new candidate links, the number of iterations needed would be greater.

To determine whether the proposed solution algorithm (DDIA) has found the optimal solution of the MNDP for this test network, the new candidate links (*u*_1_, *u*_2_, *u*_3_, *u*_4_, *u*_5_) are combined to obtain 32 total possible combinations. We solve a corresponding CNDP for each combination (Table 7).

**Table 7:** Solution under the fixed u.

u	y	*Z*
(0 0 0 0 0)	(5.25, 2.75, 5.25, 2.25, 3.25, 1.9375, 3, 5, 3.5, 5)	81.4238
(1 0 0 0 0)	(5.75, 2.25, 5.75, 3, 3, 3, 3.5, 4.5, 3.5, 4.25)	82.0686
(0 1 0 0 0)	(5.5, 3, 5, 2, 3.25, 3, 4, 5, 4, 5)	79.2225
(0 0 1 0 0)	(4, 1.5, 4, 1, 2, 2.0625, 3.5, 4, 4, 2.5)	71.8950
(0 0 0 1 0)	(4.75, 1.5, 4.75, 2.75, 3, 4, 3, 4.5, 3.25, 4.25)	78.1433
(0 0 0 0 1)	(5, 1.75, 4, 1, 2, 2, 4, 4, 4, 4)	76.2573
(1 1 0 0 0)	(5.875, 2.625, 5.625, 2.75, 2.5, 2.5, 4, 5.25, 3.375, 5)	79.7942
(1 0 1 0 0)	(4, 2, 4, 1, 2.375, 3, 4, 4, 4, 4,)	72.5985
(1 0 0 1 0)	(4.75, 3, 3.875, 2, 3.25, 3.75, 3, 4.5, 3.5, 5)	78.9416
(1 0 0 0 1)	(5.75, 1.75, 5.75, 3.25, 1.75, 1.25, 3.5, 3.25, 4, 2.5)	76.9746
(0 1 1 0 0)	(4.5, 1.5, 4.625, 2, 2.5, 2, 3.5, 3, 3.5, 3)	69.5893
(0 1 0 1 0)	(4.5, 2, 3.75, 2.25, 3, 3.125, 3, 5, 4, 4.5)	75.7549
(0 1 0 0 1)	(4.875, 1.5, 4.5, 2, 2.5, 2, 4.5, 2.9375, 5, 2.5)	74.5435
(0 0 1 1 0)	(4.125, 1, 4.125, 1.875, 1.75, 0.875, 4.0625, 3, 4, 3.125)	70.6418
(0 0 1 0 1)	(4.312, 1.8125, 4.4375, 1.5, 0.0625, 1.875, 3.9375, 3, 3.5, 3)	69.5012
(0 0 0 1 1)	(3.5, 1.5, 3.5, 1, 2.9375, 3, 4, 4, 4, 4)	73.3931
(0 0 1 1 1)	(3.75, 1, 3.5, 2, 1, 1, 4, 3, 4.5, 3)	68.8601
(0 1 0 1 1)	(3.5, 2, 3.9375, 2, 3, 3.5, 4, 2, 3.875, 2)	71.3405
(0 1 1 0 1)	(4, 1.5, 4, 1, 2.25, 1, 4, 1.5, 4, 1)	67.3721
(0 1 1 1 0)	(4, 2, 3.875, 1.375, 1, 1, 4, 2.25, 4, 2.25)	68.3805
(1 0 0 1 1)	(4, 2.25, 4.5, 2, 2.75, 3, 3.75, 4, 4, 4)	74.4567
(1 0 1 0 1)	(3, 1, 4, 1, 1, 1, 4, 3, 5, 4)	70.4735
(1 0 1 1 0)	(4.5, 2, 4.5, 1, 1.5, 1, 4, 4, 5, 4)	71.6735
(1 1 0 0 1)	(5, 2, 5, 2.5, 2, 1, 4, 3, 4, 2.5)	75.0594
(1 1 0 1 0)	(4.5, 1.5, 4.5, 2.5, 2.5, 4, 4, 5, 4, 5)	76.6699
(1 1 1 0 0)	(4, 2.5, 4, 1, 3, 1.5, 3.5, 3, 4, 3.5)	70.4913
**(0 1 1 1 1)**	**(3.5**, **1.5**, **4**, **1.75**, **1.25**, **0.75**, **4**, **1.5**, **4**, **2)**	**66.8953**
(1 0 1 1 1)	(3.5, 2.5, 4, 1.5, 2, 0.25, 4.5, 3.5, 4, 3.5)	70.0483
(1 1 0 1 1)	(4, 2, 4, 2.5, 3, 3, 4, 2, 4, 1.75)	72.5345
(1 1 1 0 1)	(3.25, 1.5, 4, 1.0625, 1.75, 1.75, 3.5625, 2, 4, 2)	68.1968
(1 1 1 1 0)	(4.5, 1.5, 5, 1.5, 1.5, 1, 4, 3, 4, 2)	69.1941
(1 1 1 1 1)	(3.5, 1.5, 4, 1.5, 0.75, 1, 4, 2, 4, 1)	67.9265

In Table 7, the solution with a minimal objective function value is **u** = (0 1 1 1 1), **y** = (3.5000 1.5000 4.0000 1.7500 1.2500 0.7500 4.0000 1.5000 4.0000 2.0000), *Z =* 66.8953, which is consistent with the solution found by the proposed solution algorithm (DDIA) (the error is 0.0378%, which arises from different initial values taken in the calculation).

The above analysis is for the case without budget constraint. We now discuss the case with budget constraint. Suppose that the total budget is 4000. Here, the DNDP is solved using the implicit enumeration method [47], and the Hooke-Jeeves algorithm is applied to solve the CNDP. To solve the CNDP with budget constraint, the penalty function method [48] is first used to transform it into an extremum problem without constraints. The results obtained for different initial values (u^(0)^) are in Tables 8~10.

**Table 8:** Results when u^(0)^ = (0 0 0 0 0).

Iteration		Fixed value	Solution	*Z*
1	solving CNDP	u^(0)^ = (0 0 0 0 0)	y^(0)^ = (3.9375 1.6875 4.3125 2.9375 2.5000 2.1875 2.9375 4.0000 4.2500 3.8750)	78.0074
2	solving DNDP	y^(0)^ = (3.9375 1.6875 4.3125 2.9375 2.5000 2.1875 2.9375 4.0000 4.2500 3.8750)	u^(1)^ = (0 0 0 0 0)	78.0074; *convergence*

**Table 9:** Results when u^(0)^ = (0 0 0 0 1).

Iteration		Fixed value	Solution	*Z*
1	solving CNDP	u^(0)^ = (0 0 0 0 1)	y^(0)^ = (3.1250 1.8750 3.0000 1.5000 2.5000 2.2500 3.0000 2.0000 2.0000 2.0000)	74.0121
2	solving DNDP	y^(0)^ = (3.1250 1.8750 3.0000 1.5000 2.5000 2.2500 3.0000 2.0000 2.0000 2.0000)	u^(1)^ = (0 0 1 0 0)	69.2053
3	solving CNDP	u^(1)^ = (0 0 1 0 0)	y^(1)^ = (4.6250 1.8750 3.0000 1.5000 2.2500 2.2500 3.0000 2.0000 2.0000 2.0000)	**68.6234**
4	solving DNDP	y^(1)^ = (4.6250 1.8750 3.0000 1.5000 2.2500 2.2500 3.0000 2.0000 2.0000 2.0000)	u^(2)^ = (0 0 1 0 0)	**68.6234;** *convergence*

**Table 10:** Results when u^(0)^ = (1 0 0 0 1).

Iteration		Fixed value	Solution	*Z*
1	solving CNDP	u^(0)^ = (1 0 0 0 1)	y^(0)^ = (1.6250 0.1875 1.5000 0.7500 0.1250 1.3750 1.2500 1.1250 1.0000 1.0000)	78.7804
2	solving DNDP	y^(0)^ = (1.6250 0.1875 1.5000 0.7500 0.1250 1.3750 1.2500 1.1250 1.0000 1.0000)	u^(1)^ = (0 1 1 0 0)	70.0644
3	solving CNDP	u^(1)^ = (0 1 1 0 0)	y^(1)^ = (2.1250 1.3125 1.5000 0.7500 1.1250 1.3750 1.2500 1.1250 1.0000 1.0000)	69.2706
4	solving DNDP	y^(1)^ = (2.1250 1.3125 1.5000 0.7500 1.1250 1.3750 1.2500 1.1250 1.0000 1.0000)	u^(2)^ = (0 1 1 0 0)	69.2706; *convergence*

The above results show that for the MNDP with budget constraint, the resulting solution depends on the selection of initial values. This leads to different optimal solutions (i.e., different local optimal solutions). Thus, for the MNDP with budget constraint, multiple initial values need to be tested and the best solution is selected from the local solutions. Here, the method of selecting the initial value **u**^**(0)**^ is as follows: within the budget constraint, several different **u**^**(0)**^ are randomly taken such that they are scattered throughout the solution space. To compare the optimal solution with the result in [15], see Table 11.

**Table 11:** Comparison of results of different methods.

Node *i*	Node *j*	LMILP	DDIA
Expanded links			
6	8	3.173	4.6250
7	8	1.084	1.8750
8	6	2.919	3.0000
8	7	1.078	1.5000
9	10	1.316	2.2500
10	9	1.564	2.2500
10	16	3.232	3.0000
13	24	2.867	2.0000
16	10	3.232	2.0000
24	13	2.638	2.0000
Potential new links			
7	16	-	-
9	11	-	-
11	9	-	-
11	15	√	√
13	14	-	-
14	13	-	-
15	11	√	√
16	7	-	-
19	22	-	-
22	19	-	-
Total travel time (10^3^ vehicle hours)			
*Z*		68.1955	68.6234
*Z’*		67.2430	
Improvement cost		4,000,000	4,000,000

Note: “√” = addition of new links.

*Z’* is the original value of objective function reported in the previous studies.

The solution obtained using LMILP in Table 11 is put into the user equilibrium assignment model (UE) and yields *Z =* 68.1955; the result in [15], where a different calculation method (piece-wise linear approximation) is applied, is 67.2430. The optimized objective function value using the proposed algorithm (DDIA) is *Z* = 68.6234, so the error (compared to 68.1955) is 0.627%. This result is preferable because the error is very small. Compared to LMILP, DDIA is very simple in theory and has a simpler calculation process (LMILP needs many piece-wise linear one- and two-dimensional approximations). The calculation time of DDIA depends on the use of algorithms for CNDPs and DNDPs. However, the linearization processing of the link impedance function in LMILP is complex and time-consuming before the calculation.

In practice, the budgets of new projects and reconstruction projects can be considered separately. For this example, suppose the budgets of new projects and reconstruction projects are both 2000. The results are shown in Table 12.

**Table 12:** The results when the budgets of new projects and reconstruction projects are both 2000.

Iteration		Fixed value	Solution	*Z*
1	solving CNDP	u^(0)^ = (0 0 0 0 0)	y^(0)^ = (2.7500 1.6250 4.0000 2.0000 2.5000 1.5000 2.7500 2.3750 2.0000 2.1250)	82.2871
2	solving DNDP	y^(0)^ = (2.7500 1.6250 4.0000 2.0000 2.5000 1.5000 2.7500 2.3750 2.0000 2.1250)	u^(1)^ = (0 0 1 0 0)	69.1590
3	solving CNDP	u^(1)^ = (0 0 1 0 0)	y^(1)^ = (2.6250 1.6250 4.0000 2.1250 2.5000 1.5000 2.7500 2.3750 2.0000 2.1250)	69.1341
4	solving DNDP	y^(1)^ = (2.6250 1.6250 4.0000 2.1250 2.5000 1.5000 2.7500 2.3750 2.0000 2.1250)	u^(2)^ = (0 0 1 0 0)	69.1341; *convergence*

Table 13 gives the solutions (**u, y**, *Z*) under different budgets for new projects (*b*_1_) and reconstruction projects (*b*_2_). It can be seen from the table that when *b*_1_/*b*_2_ is 3500/500, 2500/1500, or 2000/2000, a better solution is obtained. Compare the results for *b*_1_/*b*_2_ being 2500/1500 or 2000/2000: they have the same **u**, which shows that their real expenses on new projects are the same (the budget of new projects (*b*_1_) has a bigger surplus when *b*_1_/*b*_2_ is 2500/1500), whereas the objective function values have a very small difference (69.9314/ 69.1341). Thus, from the view of saving money, *b*_1_/*b*_2_ being 2500/1500 is better (although its corresponding *Z* is slightly larger). When *b*_1_ is less than 1500, this budget is not sufficient for any one new project, so the last *b*_1_/*b*_2_ is 0/4000.

**Table 13:** The results under different *b*_1_/*b*_2._

*b*_1_/*b*_2_	u	y	*Z*
4000/0	(0 0 1 0 1)	(0 0 0 0 0 0 0 0 0 0)	75.9316
3500/500	(0 1 1 0 0)	(1.6250 0.3125 1.5000 0.5000 0.7500 0.9375 1.1250 1.5000 1.0000 2.0000)	69.3925
3000/1000	(0 0 1 0 0)	(2.1875 0 2.0000 0.2500 1.9375 0.6250 2.0000 2.0000 2.0000 2.0625)	71.4529
2500/1500	(0 0 1 0 0)	(2.5000 1.1250 2.7500 1.6250 2.0000 2.0000 2.2500 2.2500 2.0000 2.2500)	69.9314
2000/2000	(0 0 1 0 0)	(2.6250 1.6250 4.0000 2.1250 2.5000 1.5000 2.7500 2.3750 2.0000 2.1250)	69.1341
1500/2500	(1 0 0 0 0)	(3.0000 2.2500 3.7500 3.0000 2.3750 1.8750 3.0000 3.0000 2.1250 2.0000)	80.6477
0/4000	(0 0 0 0 0)	(3.9375 1.6875 4.3125 2.9375 2.5000 2.1875 2.9375 4.0000 4.2500 3.8750)	78.0074

By giving multiple groups of *b*_1_/*b*_2_, as in Table 13, at least a non-inferior solution can be obtained. When *b*_1_/*b*_2_ is divided more carefully, the solution is closer to the optimal solution.

## Conclusion and Discussion

This paper proposed an optimization algorithm, the dimension-down iterative algorithm (DDIA), for solving the mixed transportation network design problem (MNDP). The idea of the proposed solution algorithm is to reduce the dimensions of the problem. A group of variables (discrete/continuous) is fixed to optimize another group of variables (continuous/discrete) alternately; then, the problem is transformed into solving a series of CNDPs and DNDPs repeatedly until it converges to an optimal solution. The advantage of the proposed algorithm is that its calculation process is very simple and that it can utilize existing algorithms for the CNDP and DNDP in the solution process.

It can be seen from two numerical examples (one is a simple test network, and the other is the Sioux Falls network) that for the MNDP without budget constraint, the global optimal solution can be found within a few iterations using the proposed algorithm, and the resulting solution is not affected by the initial values. However, for the MNDP with budget constraint, the result depends on the selection of initial values; therefore, for the MNDP with budget constraint, multiple initial values need to be tested. A comparison with the previously proposed methods shows that the DDIA is effective.

MNDPs involve both discrete and continuous variables and are very difficult to solve. As presented in other studies [12–14], a heuristic algorithm called DDIA is proposed in this paper. By using this algorithm, we can easily find a non-inferior solution to the problem. This may provide a reliable input for future algorithm improvements.

## Supporting Information

S1 Table Data of Sioux Falls network for MNDP. (DOC)

S2 Table Travel demand matrix for the Sioux Falls network. (DOC)

## References

[1] Yang H, Bell MGH (1998). Models and algorithms for road network design: a review and some new developments. *Transport Reviews* 18: 257–278.

[2] Patriksson M (2008). On the applicability and solution of bilevel optimization models in transportation science: a study on the existence, stability and computation of optimal solutions to stochastic mathematical programs with equilibrium constraints. *Transportation Research Part B* 42: 843–860.

[3] Liu H, Wang DZW (2015). Global optimization method for network design problem with stochastic user equilibrium. *Transportation Research Part B* 72: 20–39.

[4] Friesz TL, Hsun-jung C, Mehta NJ, Tobin RL, Anandalingam G (1992). A simulated annealing approach to the network design problem with variational inequality constraints. *Transportation Science* 26: 18–26.

[5] Meng Q, Yang H, Bell MGH (2001). An equivalent continuously differentiable model and a locally convergent algorithm for the continuous network design problem. *Transportation Research Part B* 35: 83–105.

[6] Chiou SW (2005). Bilevel programming for the continuous transport network design problem. *Transportation Research Part B* 39: 361–383.

[7] Li C, Yang H, Zhu D Meng Q. (2012). A global optimization method for continuous network design problems. *Transportation Research Part B* 46: 1144–1158.

[8] LeBlanc LJ (1975). An algorithm for the discrete network design problem. *Transportation Science* 9: 183–199.

[9] Chen MY, Alfa AS (1991). A network design algorithm using a stochastic incremental traffic assignment approach. *Transportation Science* 25: 214–224.

[10] Drezner Z, Wesolowsky GO (1997). Selecting an optimum configuration of one- way and two-way routes. *Transportation Science* 31: 386–394.

[11] Farvaresh H, Sepehri MM (2013). A branch and bound algorithm for bi-level discrete network design problem. *Networks & Spatial Economics* 13: 67–106.

[12] Cantarella GE, Pavone G, Vitetta A (2006). Heuristics for urban road network design: lane layout and signal settings. *European Journal of Operational Research* 175: 1682–1695.

[13] Dimitriou L, Tsekeris T, Stathopoulos A (2008). Genetic computation of road network design and pricing Stackelberg games with multi-class users In: Giacobini M. et al (Eds.), Applications of Evolutionary Computing. Springer, Berlin, Heidelberg, pp. 669–678.

[14] Gallo M, D' Acierno L, Montella B (2010). A meta-heuristic approach for solving the urban network design problem. *European Journal of Operational Research* 201: 144–157.

[15] Luathep P, Sumalee A, Lam WHK, Li Z, Lo HK (2011). Global optimization method for mixed transportation network design problem: A mixed-integer linear programming approach. *Transportation Research Part B* 45: 808–827.

[16] Connors RD, Sumalee A, Watling DP (2007). Sensitivity analysis of the variable demand probit stochastic user equilibrium with multiple user-classes. *Transportation Research Part B* 41: 593–615.

[17] Sumalee A, Watling DP, Nakayama S (2006). Reliable network design problem: case with uncertain demand and total travel time reliability. *Transportation Research Record* 1964: 81–90.

[18] Yang H, Yagar S (1995). Traffic assignment and signal control in saturated road networks. *Transportation Research Part A* 29: 125–139.

[19] Gao Z, Song YF (2002). A reserve capacity model of optimal signal control with user-equilibrium route choice. *Transportation Research Part B* 36: 313–323.

[20] Gao Z, Sun H, Shan L (2004). A continuous equilibrium network design model and algorithm for transit systems. *Transportation Research Part B* 38: 235–250.

[21] Wong SC, Yang H (1997). Reserve capacity of a signal-controlled road network. *Transportation Research Part B* 31: 397–402.

[22] Chiou SW (1999). Optimization of area traffic control for equilibrium network flows. *Transportation Science* 33: 279–289.

[23] Friesz TL, Tobin RL, Cho HJ, Mehta NJ (1990). Sensitivity analysis based heuristic algorithms for mathematical programs with variational inequality constraints. *Mathematical Programming* 48: 265–284.

[24] Yang H, Yagar S (1994). Traffic assignment and traffic control in general freeway-arterial corridor systems. *Transportation Research Part B* 28: 463–486.

[25] Yang H, Yagar S, Iida Y, Asakura Y (1994). An algorithm for the inflow control problem on urban freeway networks with user-optimal flows. *Transportation Research Part B* 28: 123–139.

[26] Allsop, R.E. (1974). Some possibilities of using traffic control to influence trip distribution and route choice. In: Buckley, D.J. (Ed.), Proceedings of the 6th International Symposium on Transportation and Traffic Theory. Elsevier, Amsterdam, pp. 345–374.

[27] Marcotte P (1986). Network design problem with congestion effects: a case of bi-level programming. *Mathematical Programming* 34: 142–162.

[28] Friesz TL, Harker PT (1985). Properties of the iterative optimization-equilibrium algorithm. *Civil Engineering Systems* 2: 142–154.

[29] Marcotte P, Marquis G (1992). Efficient implementation of heuristic for the continuous network design problems. *Annals of Operation Research* 34: 163–176.

[30] Abdulaal M, LeBlanc LJ (1979). Continuous equilibrium network design models. *Transportation Research Part B* 13: 19–32.

[31] Suwansirikul C, Friesz TL, Tobin RL (1987). Equilibrium decomposed optimization: a heuristic for the continuous equilibrium network design problems.*Transportation Science* 21: 254–263.

[32] Sumalee A (2007). Multi-concentric optimal charging cordon design. *Transportmetrica* 3: 41–71.

[33] Wang DZW, Lo HK (2010). Global optimum of the linearized network design problem with equilibrium flows. *Transportation Research Part B* 44: 482–492.

[34] Poorzahedy H, Turnquist MA (1982). Approximate algorithms for the discrete network design problem. *Transportation Research Part B* 16: 45–55.

[35] Magnanti TL, Wong RT (1984). Network design and transportation planning: models and algorithms. *Transportation Science* 18: 1–55.

[36] Solanki RS, Gorti JK, Southworth F (1998). Using decomposition in large-scale highway network design with a quasi-optimization heuristic.*Transportation Research Part B* 32: 127–140.

[37] Gao Z, Wu J, Sun H (2005). Solution algorithm for the bi-level discrete network design problem. *Transportation Research Part B* 39: 479–495.

[38] Poorzahedy H, Abulghasemi F (2005). Application of ant system to network design problem. *Transportation* 32: 251–273.

[39] Drezner Z, Wesolowsky GO (2003). Network design: selection and design of links and facility location. *Transportation Research Part A* 37: 241–256.

[40] Wu J, Lu H, Yu X (2012). Genetic Algorithm for Multiuser Discrete Network Design Problem under Demand Uncertainty. *Mathematical Problems in Engineering* 686272

[41] Poorzahedy H, Rouhani OM (2007). Hybrid meta-heuristic algorithms for solving network design problem. *European Journal of Operational Research* 182: 578–596.

[42] Haas I, Bekhor S (2016). A parsimonious heuristic for the discrete network design problem. *Transportmetrica A-Transport Science* 12: 43–64.

[43] Wang S, Meng Q, Yang H (2013). Global optimization methods for the discrete network design problem. *Transportation Research Part B* 50: 42–60

[44] Wang DZW, Liu H, Szeto WY (2015). A novel discrete network design problem formulation and its global optimization solution algorithm. *Transportation Research Part E* 79: 213–230.

[45] Riemann R, Wang DZW, Busch F (2015). Optimal location of wireless charging facilities for electric vehicles: flow-capturing location model with stochastic user equilibrium. *Transportation Research Part C* 58: 1–12.

[46] Sheffi Y (1985). *Urban Transportation Networks*: *Equilibrium Analysis with Mathematical Programming Methods*. Prentice-Hall, Englewood Cliffs, NJ, USA.

[47] Liu C (2001). *Modern Transportation Planning*. Renmin Jiaotong Press, Beijing.

[48] Mokhtar SB, Shetty CM (1979). *Nonlinear Programming*: *Theory and Algorithms*. John Wiley & Sons, Inc., New York.

