# Supplementary material for: An Algorithm for the Mixed Transportation Network Design Problem
Source: PLoS One. 2016 Sep 14;11(9):e0162618. doi: 10.1371/journal.pone.0162618 (PMC5023175; doi:10.1371/journal.pone.0162618)
Supplement: S1 Table — (DOC) [file pone.0162618.s001.doc]

**S1 Table**. Data of Sioux Falls network for MNDP.

| $t_{a}=t_{a}(x_{a},y_{a})=\alpha_{a}+\beta_{a}(x_{a}/(y_{a}^{0}+y_{a}))^{4},\text{ }a\in\text{A}_{2}$; $t_{a}=t_{a}(x_{a},y_{a}^{'})=\alpha_{a}+\beta_{a}(x_{a}/y_{a}^{'})^{4},\text{ }a\in\text{A}_{3}$; $Z=\sum_{a\in\text{A}_{1}\cup\text{A}_{2}\cup\text{A}_{3}} x_{a}t_{a}+0.001\sum_{a\in\text{A}_{2}} c_{a}y_{a}^{2}+0.001\sum_{a\in\text{A}_{3}} d_{a}u_{a}$ | | | | | | | |
| --- | --- | --- | --- | --- | --- | --- | --- |
| link | node | | $y_{a}^{0}$or$y_{a}^{'}$/10^3^ vehicles/hour | $\alpha_{a}$/h | $\beta_{a}$/h | $c_{a}$or$d_{a}$/ ＄10^3^ | **y** or **u** |
|  | *i* | *j* |  |  |  |  |  |
| **1** | 1 | 2 | 25.9002 | 0.06 | 0.009 |  |  |
| **2** | 1 | 3 | 23.40347 | 0.04 | 0.006 |  |  |
| **3** | 2 | 1 | 25.9002 | 0.06 | 0.009 |  |  |
| **4** | 2 | 6 | 4.95818 | 0.05 | 0.0075 |  |  |
| **5** | 3 | 1 | 23.40347 | 0.04 | 0.006 |  |  |
| **6** | 3 | 4 | 17.11052 | 0.04 | 0.006 |  |  |
| **7** | 3 | 12 | 23.40347 | 0.04 | 0.006 |  |  |
| **8** | 4 | 3 | 17.11052 | 0.04 | 0.006 |  |  |
| **9** | 4 | 5 | 17.78279 | 0.02 | 0.003 |  |  |
| **10** | 4 | 11 | 4.90883 | 0.06 | 0.009 |  |  |
| **11** | 5 | 4 | 17.78279 | 0.02 | 0.003 |  |  |
| **12** | 5 | 6 | 4.948 | 0.04 | 0.006 |  |  |
| **13** | 5 | 9 | 10 | 0.05 | 0.0075 |  |  |
| **14** | 6 | 2 | 4.95818 | 0.05 | 0.0075 |  |  |
| **15** | 6 | 5 | 4.948 | 0.04 | 0.006 |  |  |
| **16** | 6 | 8 | 4.89859 | 0.02 | 0.003 | 26 | *y*_1_ |
| **17** | 7 | 8 | 7.84181 | 0.03 | 0.0045 | 40 | *y*_2_ |
| **18** | 7 | 18 | 23.40347 | 0.02 | 0.003 |  |  |
| **19** | 8 | 6 | 4.89859 | 0.02 | 0.003 | 26 | *y*_3_ |
| **20** | 8 | 7 | 7.84181 | 0.03 | 0.0045 | 40 | *y*_4_ |
| **21** | 8 | 9 | 5.05019 | 0.1 | 0.015 |  |  |
| **22** | 8 | 16 | 5.04582 | 0.05 | 0.0075 |  |  |
| **23** | 9 | 5 | 10 | 0.05 | 0.0075 |  |  |
| **24** | 9 | 8 | 5.05019 | 0.1 | 0.015 |  |  |
| **25** | 9 | 10 | 13.91579 | 0.03 | 0.0045 | 25 | *y*_5_ |
| **26** | 10 | 9 | 13.91579 | 0.03 | 0.0045 | 25 | *y*_6_ |
| **27** | 10 | 11 | 10 | 0.05 | 0.0075 |  |  |
| **28** | 10 | 15 | 13.512 | 0.06 | 0.009 |  |  |
| **29** | 10 | 16 | 5.1335 | 0.05 | 0.0075 | 48 | *y*_7_ |
| **30** | 10 | 17 | 4.99351 | 0.08 | 0.012 |  |  |
| **31** | 11 | 4 | 4.90883 | 0.06 | 0.009 |  |  |
| **32** | 11 | 10 | 10 | 0.05 | 0.0075 |  |  |
| **33** | 11 | 12 | 4.90883 | 0.06 | 0.009 |  |  |
| **34** | 11 | 14 | 4.87651 | 0.04 | 0.006 |  |  |
| **35** | 12 | 3 | 23.40347 | 0.04 | 0.006 |  |  |
| **36** | 12 | 11 | 4.90883 | 0.06 | 0.009 |  |  |
| **37** | 12 | 13 | 25.9002 | 0.03 | 0.0045 |  |  |
| **38** | 13 | 12 | 25.9002 | 0.03 | 0.0045 |  |  |
| **39** | 13 | 24 | 5.09126 | 0.04 | 0.006 | 34 | *y*_8_ |
| **40** | 14 | 11 | 4.87651 | 0.04 | 0.006 |  |  |
| **41** | 14 | 15 | 5.12753 | 0.05 | 0.0075 |  |  |
| **42** | 14 | 23 | 4.92479 | 0.04 | 0.006 |  |  |
| **43** | 15 | 10 | 13.512 | 0.06 | 0.009 |  |  |
| **44** | 15 | 14 | 5.12753 | 0.05 | 0.0075 |  |  |
| **45** | 15 | 19 | 15.6508 | 0.04 | 0.006 |  |  |
| **46** | 15 | 22 | 10.315 | 0.04 | 0.006 |  |  |
| **47** | 16 | 8 | 5.04582 | 0.05 | 0.0075 |  |  |
| **48** | 16 | 10 | 5.1335 | 0.05 | 0.0075 | 48 | *y*_9_ |
| **49** | 16 | 17 | 5.22991 | 0.02 | 0.003 |  |  |
| **50** | 16 | 18 | 19.6799 | 0.03 | 0.0045 |  |  |
| **51** | 17 | 10 | 4.99351 | 0.08 | 0.012 |  |  |
| **52** | 17 | 16 | 5.22991 | 0.02 | 0.003 |  |  |
| **53** | 17 | 19 | 4.82395 | 0.02 | 0.003 |  |  |
| **54** | 18 | 7 | 23.40347 | 0.02 | 0.003 |  |  |
| **55** | 18 | 16 | 19.6799 | 0.03 | 0.0045 |  |  |
| **56** | 18 | 20 | 23.40347 | 0.04 | 0.006 |  |  |
| **57** | 19 | 15 | 15.6508 | 0.04 | 0.006 |  |  |
| **58** | 19 | 17 | 4.82395 | 0.02 | 0.003 |  |  |
| **59** | 19 | 20 | 5.00261 | 0.04 | 0.006 |  |  |
| **60** | 20 | 18 | 23.40347 | 0.04 | 0.006 |  |  |
| **61** | 20 | 19 | 5.00261 | 0.04 | 0.006 |  |  |
| **62** | 20 | 21 | 5.05991 | 0.06 | 0.009 |  |  |
| **63** | 20 | 22 | 5.0757 | 0.05 | 0.0075 |  |  |
| **64** | 21 | 20 | 5.05991 | 0.06 | 0.009 |  |  |
| **65** | 21 | 22 | 5.22991 | 0.02 | 0.003 |  |  |
| **66** | 21 | 24 | 4.88536 | 0.03 | 0.0045 |  |  |
| **67** | 22 | 15 | 10.315 | 0.04 | 0.006 |  |  |
| **68** | 22 | 20 | 5.0757 | 0.05 | 0.0075 |  |  |
| **69** | 22 | 21 | 5.22991 | 0.02 | 0.003 |  |  |
| **70** | 22 | 23 | 5 | 0.04 | 0.006 |  |  |
| **71** | 23 | 14 | 4.92479 | 0.04 | 0.006 |  |  |
| **72** | 23 | 22 | 5 | 0.04 | 0.006 |  |  |
| **73** | 23 | 24 | 5.07851 | 0.02 | 0.003 |  |  |
| **74** | 24 | 13 | 5.09126 | 0.04 | 0.006 | 34 | *y*_10_ |
| **75** | 24 | 21 | 4.88536 | 0.03 | 0.0045 |  |  |
| **76** | 24 | 23 | 5.07851 | 0.02 | 0.003 |  |  |
| **77** | 7 | 16 | 10.8812 | 0.03 | 0.0045 | 750 | *u*_1_ |
| **78** | 16 | 7 | 10.8812 | 0.03 | 0.0045 | 750 | *u*_1_ |
| **79** | 19 | 22 | 13.74708 | 0.01 | 0.0015 | 825 | *u*_2_ |
| **80** | 22 | 19 | 13.74708 | 0.01 | 0.0015 | 825 | *u*_2_ |
| **81** | 11 | 15 | 8.60172 | 0.015 | 0.00225 | 900 | *u*_3_ |
| **82** | 15 | 11 | 8.60172 | 0.015 | 0.00225 | 900 | *u*_3_ |
| **83** | 9 | 11 | 18.4008 | 0.0214 | 0.00321 | 975 | *u*_4_ |
| **84** | 11 | 9 | 18.4008 | 0.0214 | 0.00321 | 975 | *u*_4_ |
| **85** | 13 | 14 | 9.83995 | 0.01 | 0.0015 | 1050 | *u*_5_ |
| **86** | 14 | 13 | 9.83995 | 0.01 | 0.0015 | 1050 | *u*_5_ |
