# Supplementary material for: An Algorithm for the Mixed Transportation Network Design Problem
Source: PLoS One. 2016 Sep 14;11(9):e0162618. doi: 10.1371/journal.pone.0162618 (PMC5023175; doi:10.1371/journal.pone.0162618)
Supplement: S2 Table — (DOC) [file pone.0162618.s002.doc]

**S2 Table. Travel demand** matrix for the Sioux Falls network.

| **node** | **1** | **2** | **3** | **4** | **5** | **6** | **7** | **8** | **9** | **10** | **11** | **12** | **13** | **14** | **15** | **16** | **17** | **18** | **19** | **20** | **21** | **22** | **23** | **24** |
| --- | --- | --- | --- | --- | --- | --- | --- | --- | --- | --- | --- | --- | --- | --- | --- | --- | --- | --- | --- | --- | --- | --- | --- | --- |
| **1** | **0** | **0.11** | **0.11** | **0.55** | **0.22** | **0.33** | **0.55** | **0.88** | **0.55** | **1.43** | **0.55** | **0.22** | **0.55** | **0.33** | **0.55** | **0.55** | **0.44** | **0.11** | **0.33** | **0.33** | **0.11** | **0.44** | **0.33** | **0.11** |
| **2** | **0.11** | **0** | **0.11** | **0.22** | **0.11** | **0.44** | **0.22** | **0.44** | **0.22** | **0.66** | **0.22** | **0.11** | **0.33** | **0.11** | **0.11** | **0.44** | **0.22** | **0** | **0.11** | **0.11** | **0** | **0.11** | **0** | **0** |
| **3** | **0.11** | **0.11** | **0** | **0.22** | **0.11** | **0.33** | **0.11** | **0.22** | **0.11** | **0.33** | **0.33** | **0.22** | **0.11** | **0.11** | **0.11** | **0.22** | **0.11** | **0** | **0** | **0** | **0** | **0.11** | **0.11** | **0** |
| **4** | **0.55** | **0.22** | **0.22** | **0** | **0.55** | **0.44** | **0.44** | **0.77** | **0.77** | **1.32** | **1.54** | **0.66** | **0.66** | **0.55** | **0.55** | **0.88** | **0.55** | **0.11** | **0.22** | **0.33** | **0.22** | **0.44** | **0.55** | **0.22** |
| **5** | **0.22** | **0.11** | **0.11** | **0.55** | **0** | **0.22** | **0.22** | **0.55** | **0.88** | **1.1** | **0.55** | **0.22** | **0.22** | **0.11** | **0.22** | **0.55** | **0.22** | **0** | **0.11** | **0.11** | **0.11** | **0.22** | **0.11** | **0** |
| **6** | **0.33** | **0.44** | **0.33** | **0.44** | **0.22** | **0** | **0.44** | **0.88** | **0.44** | **0.88** | **0.44** | **0.22** | **0.22** | **0.11** | **0.22** | **0.99** | **0.55** | **0.11** | **0.22** | **0.33** | **0.11** | **0.22** | **0.11** | **0.11** |
| **7** | **0.55** | **0.22** | **0.11** | **0.44** | **0.22** | **0.44** | **0** | **1.1** | **0.66** | **2.09** | **0.55** | **0.77** | **0.44** | **0.22** | **0.55** | **1.54** | **1.1** | **0.22** | **0.44** | **0.55** | **0.22** | **0.55** | **0.22** | **0.11** |
| **8** | **0.88** | **0.44** | **0.22** | **0.77** | **0.55** | **0.88** | **1.1** | **0** | **0.88** | **1.76** | **0.88** | **0.66** | **0.66** | **0.44** | **0.66** | **2.42** | **1.54** | **0.33** | **0.77** | **0.99** | **0.44** | **0.55** | **0.33** | **0.22** |
| **9** | **0.55** | **0.22** | **0.11** | **0.77** | **0.88** | **0.44** | **0.66** | **0.88** | **0** | **3.08** | **1.54** | **0.66** | **0.66** | **0.66** | **0.99** | **1.54** | **0.99** | **0.22** | **0.44** | **0.66** | **0.33** | **0.77** | **0.55** | **0.22** |
| **10** | **1.43** | **0.66** | **0.33** | **1.32** | **1.1** | **0.88** | **2.09** | **1.76** | **3.08** | **0** | **4.4** | **2.2** | **2.09** | **2.31** | **4.4** | **4.84** | **4.29** | **0.77** | **1.98** | **2.75** | **1.32** | **2.86** | **1.98** | **0.88** |
| **11** | **0.55** | **0.22** | **0.33** | **1.65** | **0.55** | **0.44** | **0.55** | **0.88** | **1.54** | **4.29** | **0** | **1.54** | **1.1** | **1.76** | **1.54** | **1.54** | **1.1** | **0.11** | **0.44** | **0.66** | **0.44** | **1.21** | **1.43** | **0.66** |
| **12** | **0.22** | **0.11** | **0.22** | **0.66** | **0.22** | **0.22** | **0.77** | **0.66** | **0.66** | **2.2** | **1.54** | **0** | **1.43** | **0.77** | **0.77** | **0.77** | **0.66** | **0.22** | **0.33** | **0.44** | **0.33** | **0.77** | **0.77** | **0.55** |
| **13** | **0.55** | **0.33** | **0.11** | **0.66** | **0.22** | **0.22** | **0.44** | **0.66** | **0.66** | **2.09** | **1.1** | **1.43** | **0** | **0.66** | **0.77** | **0.66** | **0.55** | **0.11** | **0.33** | **0.66** | **0.66** | **1.43** | **0.88** | **0.88** |
| **14** | **0.33** | **0.11** | **0.11** | **0.55** | **0.11** | **0.11** | **0.22** | **0.44** | **0.66** | **2.31** | **1.76** | **0.77** | **0.66** | **0** | **1.43** | **0.77** | **0.77** | **0.11** | **0.33** | **0.55** | **0.44** | **1.32** | **1.21** | **0.44** |
| **15** | **0.55** | **0.11** | **0.11** | **0.55** | **0.22** | **0.22** | **0.55** | **0.66** | **1.1** | **4.4** | **1.54** | **0.77** | **0.77** | **1.43** | **0** | **1.32** | **1.65** | **0.22** | **0.88** | **1.21** | **0.88** | **2.86** | **1.1** | **0.44** |
| **16** | **0.55** | **0.44** | **0.22** | **0.88** | **0.55** | **0.99** | **1.54** | **2.42** | **1.64** | **4.84** | **1.54** | **0.77** | **0.66** | **0.77** | **1.32** | **0** | **3.08** | **0.55** | **1.43** | **1.76** | **0.66** | **1.32** | **0.55** | **0.33** |
| **17** | **0.44** | **0.22** | **0.11** | **0.55** | **0.22** | **0.55** | **1.1** | **1.54** | **0.99** | **4.29** | **1.1** | **0.66** | **0.55** | **0.77** | **1.65** | **3.08** | **0** | **0.66** | **1.87** | **1.87** | **0.66** | **1.87** | **0.66** | **0.33** |
| **18** | **0.11** | **0** | **0** | **0.11** | **0** | **0.11** | **0.22** | **0.33** | **0.22** | **0.77** | **0.22** | **0.22** | **0.11** | **0.11** | **0.22** | **0.55** | **0.66** | **0** | **0.33** | **0.44** | **0.11** | **0.33** | **0.11** | **0** |
| **19** | **0.33** | **0.11** | **0** | **0.22** | **0.11** | **0.22** | **0.44** | **0.77** | **0.44** | **1.98** | **0.44** | **0.33** | **0.33** | **0.33** | **0.88** | **1.43** | **1.87** | **0.33** | **0** | **1.32** | **0.44** | **1.32** | **0.33** | **0.11** |
| **20** | **0.33** | **0.11** | **0** | **0.33** | **0.11** | **0.33** | **0.55** | **0.99** | **0.66** | **2.75** | **0.66** | **0.55** | **0.66** | **0.55** | **1.21** | **1.76** | **1.87** | **0.44** | **1.32** | **0** | **1.32** | **2.64** | **0.77** | **0.44** |
| **21** | **0.11** | **0** | **0** | **0.22** | **0.11** | **0.11** | **0.22** | **0.44** | **0.33** | **1.32** | **0.44** | **0.33** | **0.66** | **0.44** | **0.88** | **0.66** | **0.66** | **0.11** | **0.44** | **1.32** | **0** | **1.98** | **0.77** | **0.55** |
| **22** | **0.44** | **0.11** | **0.11** | **0.44** | **0.22** | **0.22** | **0.55** | **0.55** | **0.77** | **2.86** | **1.21** | **0.77** | **1.43** | **1.32** | **2.86** | **1.32** | **1.87** | **0.33** | **1.32** | **2.64** | **1.98** | **0** | **2.31** | **1.21** |
| **23** | **0.33** | **0** | **0.11** | **0.55** | **0.11** | **0.11** | **0.22** | **0.33** | **0.55** | **1.98** | **1.43** | **0.77** | **0.88** | **1.21** | **1.1** | **0.55** | **0.66** | **0.11** | **0.33** | **0.77** | **0.77** | **2.31** | **0** | **0.77** |
| **24** | **0.11** | **0** | **0** | **0.22** | **0** | **0.11** | **0.11** | **0.22** | **0.22** | **0.88** | **0.66** | **0.55** | **0.77** | **0.44** | **0.44** | **0.33** | **0.33** | **0** | **0.11** | **0.44** | **0.55** | **1.21** | **0.77** | **0** |
